# Knowledge and attitudes about rare genetic diseases among practitioners of oral medicine/pathology in Brazil: a cross-sectional study

**DOI:** 10.3389/froh.2025.1573355

**Published:** 2025-07-07

**Authors:** Samuel Trezena, Daniella Reis Barbosa Martelli, Paulo Rogério Ferreti Bonan, Edgard Graner, Lívia Maria Ferreira Sobrinho, Faizan Alawi, Ricardo D. Coletta, Hercílio Martelli-Júnior

**Affiliations:** ^1^ Department of Dentistry, Center of Health and Biological Sciences, State University of Montes Claros—Unimontes, Montes Claros, Brazil; ^2^ Postgraduate Program in Health Sciences, Montes Claros State University—Unimontes, Montes Claros, Brazil; ^3^ Department of Clinical and Social Dentistry, Health Sciences Center, Federal University of Paraíba—UFPB, João Pessoa, Brazil; ^4^ Department of Oral Diagnosis, Faculty of Dentistry of Piracicaba, State University of Campinas—Unicamp, Piracicaba, Brazil; ^5^ Porto Alegre Clinical Hospital, Research and Postgraduate Group, Porto Alegre Clinical Hospital, Porto Alegre, Brazil; ^6^ Department of Basic & Translational Sciences, University of Pennsylvania, Penn School of Dental Medicine, Philadelphia, PA, United States

**Keywords:** genetic diseases, inborn, rare diseases, syndrome, tooth abnormalities, oral medicine, oral pathology

## Abstract

**Introduction:**

This study aimed to analyze the knowledge and attitudes of Brazilian Oral Medicine and Pathology (OM/OP) specialists about genetic diseases.

**Methods:**

A cross-sectional and descriptive study was conducted with Brazilian OM/OP specialists, using a pre-structured online formulary. Statistical analyses were performed using Statistical Package for the Social Sciences (SPSS®). The questionnaire was sent to 273 specialists, members of the Brazilian Society of Stomatology and Oral Pathology (SOBEP).

**Results:**

A total of 58 (21.2%) OM/OP specialists responded to the questionnaire. Most of the participants (67.2%) have declared attending theoretical courses on diagnosing and genetic testing for genetic diseases. Furthermore, 79.3% of participants reported that there are barriers to integration between the fields of Medical Genetics and OM/OP. Longer time working as a PhD was associated with knowledge of lesions predictive of genetic diseases (*P* < 0.05). Dental abnormalities and the presence of tumors, along with Gorlin-Goltz (nevoid basal cell carcinoma syndrome) and Gardner syndromes and neurofibromatosis, were the most frequently reported conditions and recalled by the responders of the survey.

**Conclusions:**

There is limited integration between Medical Genetics and OM/OP. However, there is considerable knowledge about oral manifestations as indicators of genetic diseases among OM/OP experts.

## 1 Introduction

Clinical genetics has revolutionized the understanding and treatment of several health conditions, especially genetic diseases and syndromes (1). Precision medicine, which aims to tailor treatments based on individual genetic, environmental and lifestyle variations, is showing great results, mostly in the treatment of rare and complex diseases (2). However, due to the reliance on genomics to develop therapies and diagnostic tests, there has been an increase in discussions regarding disparities in healthcare, particularly among populations with rare genetic variants or the presence of “orphan” diseases (2–4).

The development of therapies and management of rare genetic diseases still faces challenges. There are difficulties in attracting financial resources for research, a lack of multidisciplinary teams, and data from different populations are limited (2, 4–6). Gene therapy, which includes advanced techniques such as CRISPR-Cas9, viral vector delivery systems, and RNA-based modulation, has emerged as a transformative strategy for correcting or replacing defective genes (7, 8). For example, therapies targeting mutations in genes such as *PTCH1* (associated with Gorlin-Goltz syndrome) or *NF1* (related to neurofibromatosis type 1) underscore its therapeutic potential (9). Nevertheless, fewer than 10% of rare diseases currently have FDA-approved therapies, and diagnostic delays remain a global challenge, with an average duration of 5–7 years before a definitive diagnosis is reached (10, 11). These disparities are further pronounced in low-resource settings, where access to genetic testing and specialized care continues to be limited (12).

The integration of Medical Genetics with Oral Medicine and Pathology (OM/OP) and has been fundamental for the advancement of personalized medicine, particularly in the diagnosis of rare genetic diseases (2, 3, 6, 13). In Brazil, OM/OP specialists are responsible for the diagnosis and treatment of diseases affecting the orofacial region. In addition, the integration of genetic knowledge is necessary, allowing a more precise and personalized approach (14–16). According to the literature, many rare genetic diseases may have manifestations in teeth, oral mucosa, and structures of the head and neck region. Moreover, they may be accompanied by underlying cutaneous or systemic signs (17–26). Specific genetic conditions, such as ectodermal dysplasia, Gorlin-Goltz syndrome (also called Gorlin syndrome or nevoid basal cell carcinoma syndrome), and neurofibromatosis type 1, are examples that present orofacial alterations requiring a deep understanding (9, 20, 22) and the need for the presence of an OM/OP specialist for the multidisciplinary care (13).

Despite the critical role of OM/OP specialists, a global survey revealed that only 22% of dentists feel confident interpreting genetic test results (27). In Brazil, structural barriers, such as centralized genetic services and a shortage of trained professionals, further hinder interdisciplinary collaboration (28). Due to the scarcity of studies evaluating the relationship between clinical medical genetics and OM/OP, the present study aims to analyze the knowledge and attitudes of Brazilian OM/OP specialists about rare genetic diseases.

## 2 Material and methods

This cross-sectional and descriptive study was conducted in accordance with the STROBE (Strengthening the Reporting of Observational Studies in Epidemiology) guidelines for reporting observational research study (29). The survey was conducted from May to July 2024 with OM/OP specialists from all regions of Brazil. Convenience sampling was adopted to recruit participants. The participants were members of the Brazilian Society of Stomatology and Oral Pathology (SOBEP), Brazil.

**Figure 1:**
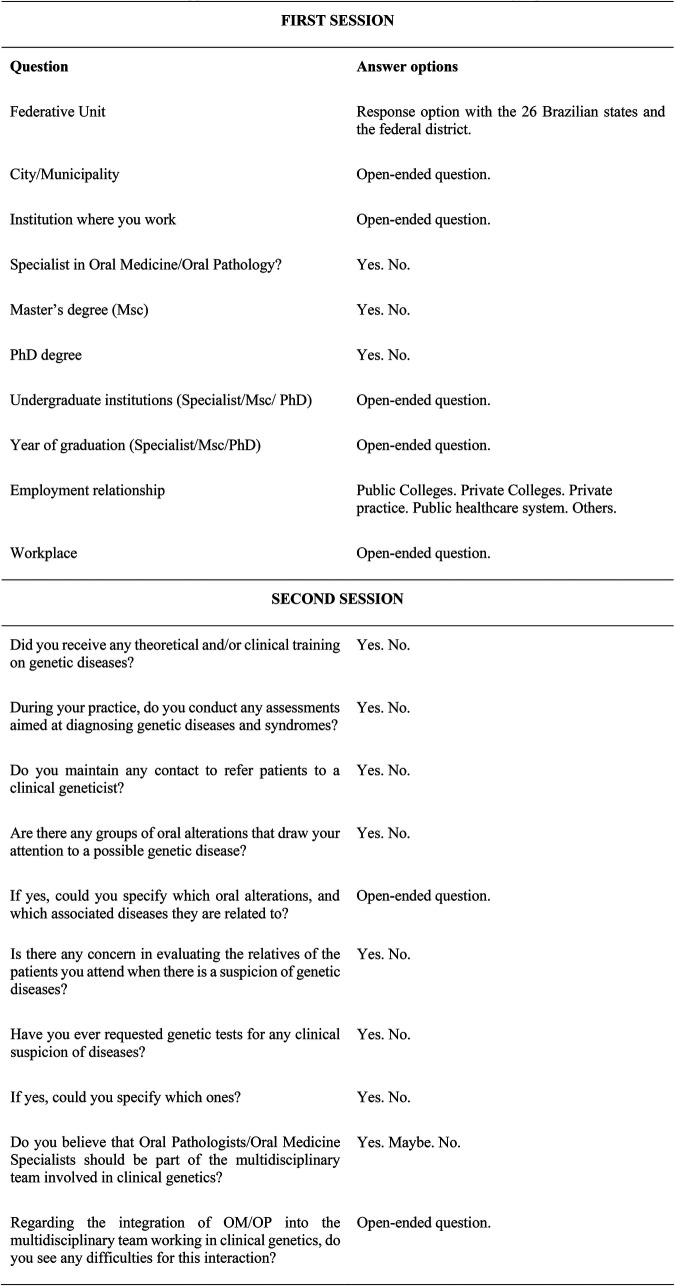
Questionnaire items applied to the of Brazilian oral medicine and pathology specialists.

The instrument was a questionnaire, available online and sent via the Google Foms® tool. The form, sent by e-mail, contained a cover letter about the study, explaining voluntary participation in the research and acceptance to participate in the study by answering the option “*I declare that I have read and agree to participate in the research”*. The questionnaire contained 14 questions divided into two sessions. The first session contained variables related to the characterization of the participants and the second session presented open and closed questions on the topic of genetic diseases (Figure 1).

The instrument was sent to 273 professionals with registered emails available on SOBEP website. Participants who did not select the agreement option were excluded. Blank and incomplete answers were also excluded from the study. The answers were automatically consolidated in Microsoft Excel spreadsheets. The spreadsheets were downloaded and exported to the Statistical Package for the Social Sciences (SPSS®) 27.0 for database construction. Descriptive analyses of frequency (n), percentile (%), mean, and standard deviation (SD) were performed. Student's t-test was used to assess the mean duration of professional experience since obtaining the degree in relation to variables associated with practice and knowledge. A *P* value of ≤0.05 was considered statistically significant. The entire study was conducted after approval of the Ethics and Research Committee (number: 78387924.0.0000.5141).

## 3 Results

**Figure 2:**
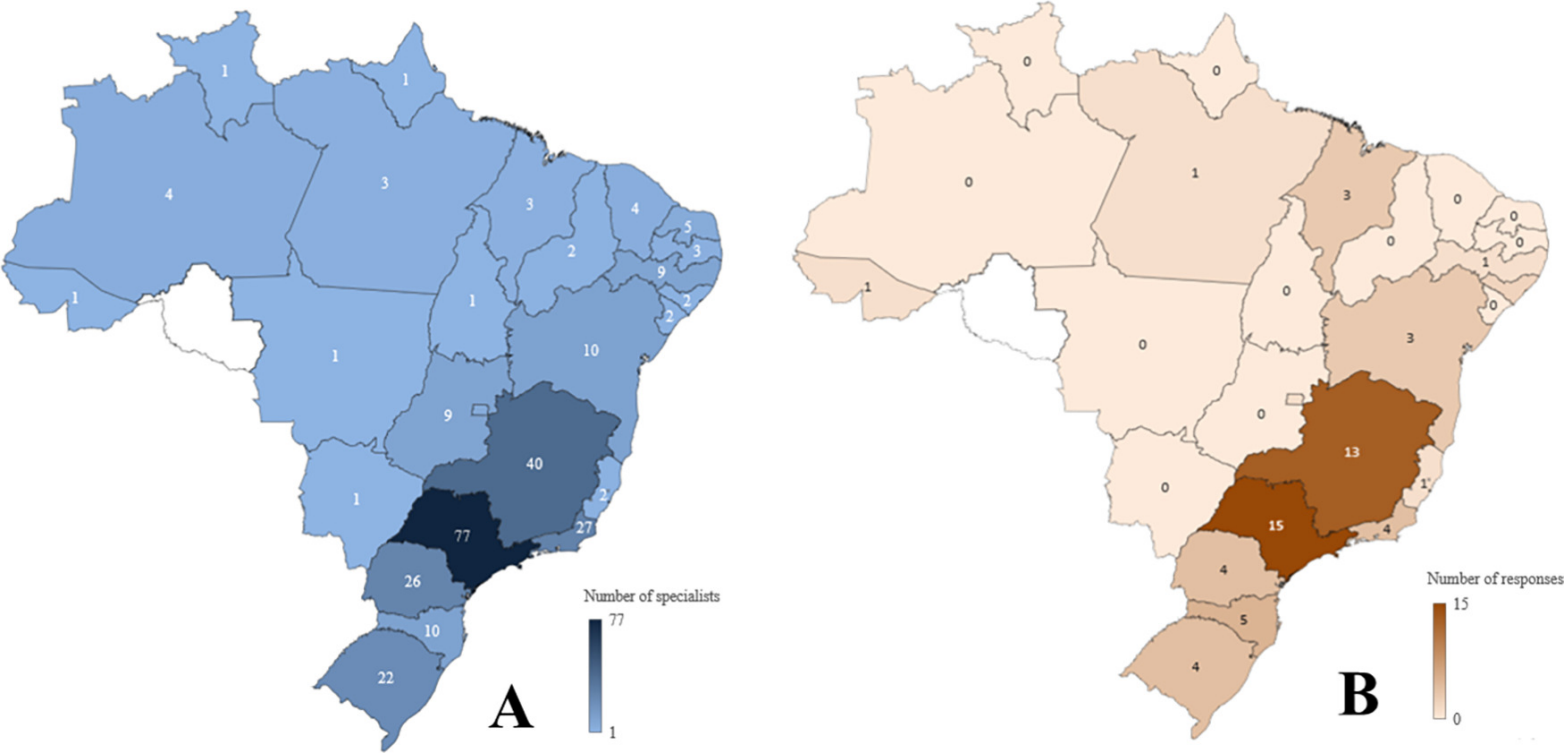
Geographical distribution of SOBEP-affiliated specialists and survey respondents by Brazilian state. **(A)** Number of specialists affiliated with the Brazilian Society of Oral Medicine and Oral Pathology (SOBEP) by state. **(B)** Number of respondents to the national survey on professional profile and knowledge related to genetic conditions with oral manifestations. Darker shades indicate higher counts in both maps.

The distribution of specialists by state and the response rate are presented in Figure 2. The state of São Paulo has the highest number of specialists (28.2%, *n* = 77); however, it accounted for 19.5% of the responses. States such as Acre, Alagoas, and Maranhão achieved a 100% response rate, but they have a low number of specialists (Range 1–3). Two SOBEP-affiliated professionals are based outside Brazil. Out of the 273 professionals, 58 (21.2%) responded to the questionnaire. Most of the participating specialists resided in the state of São Paulo (25.9%), followed by Minas Gerais (22.4%) and Santa Catarina (8.6%). The average time of practice as an OM/OP specialist was 18.4 years (SD ± 8.9 years), 18.6 years (SD ± 8.1 years) as a master, and 14.6 years (SD ± 7.7 years) as a PhD. The characteristics of professionals are depicted in Table 1.

**Table 1:** Characterization of the participants of the study (*n* = 58).

Variable	*n*	%
Brazilian Region		
North	2	3.4
Northeast	9	15.5
Midwest	1	1.8
Southeast	33	56.2
South	13	22.1
Training		
Specialist	34	58.6
Master's degree (Msc)	51	87.9
PhD degree	48	82.8
Affiliation^a^		
Public colleges	35	60.3
Private colleges	15	25.9
Private practice	19	32.8
Public healthcare system	6	10.3

^a^ Multiple-choice question

Regarding training for the diagnosis of genetic diseases, more than half of the professionals reported having received theoretical content (67.2%), and 70.7% of the professionals reported that they conduct evaluations aimed at diagnosing these diseases. Less than one-third of the professionals (24.1%) have requested genetic tests to assist in the diagnosis, and some respondents (13.8%) have doubts regarding the inclusion of OM/OP specialists in the multidisciplinary team involved in clinical genetics. A longer duration of professional experience following PhD completion (15.5 ± 7.99 years) was significantly associated (*P* = 0.022) with greater knowledge of specific groups of oral manifestations indicative of a probable diagnosis of genetic disorders.

Most participants reported that a barrier to integration between specialties with medical genetics is the lack of multidisciplinary collaboration with medicine, followed by the shortage of professionals trained for clinical genetics practice and the lack of specialized services (Table 2). Tables 3, 4 present the groups of oral alterations and probable diagnoses reported by the professionals, which are commonly observed and identified during clinical practice. The following conditions were reported once each (2%): Pierre Robin sequence; Treacher-Collins syndrome; hypophosphatasia; Kabuki syndrome; Ascher syndrome; Paget's disease; Inborn Errors of Metabolism (IEMs); Papillon-Lefèvre syndrome; and Ehlers-Danlos syndrome. Other non-genetic syndromes were also mentioned by participants.

**Table 2:** Management of the oral medicine/pathology specialists for patients with suspected genetic diseases (*n* = 58).

Variable	*n*	%
Genetic disease training		
Yes	39	67.2
No	19	32.8
Performs assessments focused on diagnosing genetic diseases		
Yes	41	70.7
No	17	29.3
Contact to refer patients to a clinical geneticist		
Yes	20	34.5
No	38	65.5
Are there specific groups of oral alterations that suggest a probable diagnosis of genetic diseases?		
Yes	49	84.5
No	9	15.5
Evaluates relatives of patients with suspected genetic diseases		
Yes	53	91.4
No	5	8.6
Requested genetic tests for suspicion of a genetic disease		
Yes	14	24.1
No	44	75.9
Oral Pathology/Oral Medicine should be part of the multidisciplinary team involved in clinical genetics		
Yes	50	86.2
Maybe	8	13.8
Difficulties in integrating Oral Pathology/Oral Medicine into the multidisciplinary team involved in clinical genetics		
Yes	46	79.3
No	12	20.7
Reasons for integration difficulties		
Lack of knowledge	9	19.5
Lack of recognition of Dentistry/Oral Medicine	13	28.2
Deficient professional education	10	21.7
Multidisciplinary deficiency	15	32.6
Lack of specialized professionals/services	14	30.4
Decreased demand	3	6.5
Low remuneration	3	6.5

**Table 3:** Oral alterations reported by the oral medicine/pathology specialists (*n* = 50).

Oral alterations	*n*	%
Dental abnormalities	26	52
Tumor presence	21	42
Facial deformities/orofacial changes	14	28
Tissue alterations	14	28
Gnathic changes	13	26
Tissue proliferation/“growth”	10	20
Pigmented lesions/spots	9	18
Ulcerations	4	8
Vascular malformations	3	6
Bleeding	2	4
Dermatological changes	2	4
Others	9	18

**Table 4:** Genetic diseases with orofacial alterations reported by the oral medicine/pathology specialists (*n* = 50).

Diagnostic hypotheses	*n*	%
Gorlin-Goltz syndrome (nevoid basal cell carcinoma syndrome)	15	30
Gardner syndrome	8	16
Neurofibromatosis	8	16
Peutz Jegher syndrome	7	14
Cleidocranial dysplasia	5	10
Cowden syndrome	4	8
Amelogenesis imperfecta with nephrocalcinosis	3	6
Apert syndrome	3	6
Crouzon syndrome	3	6
Down syndrome	3	6
McCune-Albright syndrome	3	6
Osteogenesis imperfecta	3	6
Cherubism	2	4
Crohn's disease	2	4
Epidermolysis bullosa	2	4
Fanconi anemia	2	4
Hereditary gingival hyperplasia	2	4
Mucopolysaccharidosis	2	4
Sjögren syndrome	2	4
Sturge-Weber syndrome	2	4
Other diseases	14	28

Regarding the conduct in requesting genetic tests, eight professionals reported that they refer patients to a medical team, citing reasons such as lack of knowledge, high cost of the tests, or existing partnerships with human genetics teams and services. Among the tests reported by Oral Medicine specialists were karyotyping, exome sequencing, western blot and genetic sequencing by next generation sequencing (NGS).

## 4 Discussion

The results of this study highlight the importance of integration between OM/OP and Medical Genetics, particularly in the context of the diagnosis of genetic diseases. An experience in a specialized center for rare genetic bone diseases (30), the need for a collaborative organizational model is emphasized. The presence of a multiprofessional team aimed at providing comprehensive care to patients who may present with under-investigated and complex pathologies is essential for an adequate management of the patients (30). The presence of an OM/OP specialist is recommended in multidisciplinary teams, as genetic or congenital diseases can predispose individuals to syndromes that commonly manifest with oral alterations. Furthermore, the presence of oral manifestations may be the first or most accessible manifestations and this would lead to suspicion and a more complete investigation of the patient (6, 21, 23, 24, 31–34).

Even with an average of over ten years of specialization in the field, a gap was identified in the management of patients with genetic diseases. Although a significant percentage reported having received theoretical content during their training, only 24.1% have requested, at least, a genetic test during practice. This data suggests a practical barrier to utilizing diagnostic tools. The teaching of clinical genetics in undergraduate and graduate studies dentistry should represent the training of professionals for diagnosis and appropriate management of patients with genetic diseases, as well as of their families (35). However, some countries experience deficiencies in dental genetics’ education (27, 36, 37) For example, Brazil, one of the countries that graduates the most dentists in the world, focuses its dental genetics training almost exclusively on scientific research (36). Evidenced by the results of the present study, which show that most specialists worked in the university education sector, most often in the field of scientific research.

The variables related to the participants’ perceptions in the present study align with the information discussed earlier. They identify the main barriers to integration with medical genetics as the lack of interdisciplinary collaboration with medical geneticists, followed by the shortage of professionals trained in clinical genetics. There is an urgency in the development of educational and continuing training programs, which should focus on interdisciplinary collaboration and provide practical skills for integrating genetic knowledge into the clinic (36–41). In addition, the lack of specialized services in Brazil was highlighted as a significant barrier. The Brazilian Unified Health System (SUS) established the National Policy for Comprehensive Care for People with Rare Diseases in 2014 (https://bvsms.saude.gov.br/bvs/saudelegis/gm/2014/prt0199_30_01_2014.html), which provides comprehensive care and treatment for individuals with these diseases, guidance to families, and genetic counseling (42). However, according to the literature, due to the extensive territorial dimension, lack of qualified professionals, and a healthcare network with centralized services for rare conditions, users and their caregivers often face a taxing therapeutic journey. This corroborates the fact that the number of centers for genetic diseases is still minimal compared to the actual demand (12, 28).

The perception of difficulties in integrating specialties is also reflected in the doubts of professionals about the role of OM/OP in the multidisciplinary team of clinical genetics (13.8% of respondents). There is a need for greater clarity and theoretical knowledge about the functions of these professionals in the care of patients with genetic diseases (20, 36, 42, 43). The limitation of access to essential resources for the diagnosis and management of genetic diseases hinders multidisciplinary development and integrated dental care (6, 9, 43–45). To mitigate potential risks, the establishment of reference centers and the strengthening of specialized clinical genetics services are valuable steps toward overcoming structural limitations and providing adequate support to team professionals.

The presence of dental abnormalities and tumors were the conditions reported by experts that are commonly associated with rare genetic diseases. Genetic conditions such as Gorlin-Goltz syndrome (nevoid basal cell carcinoma syndrome), Gardner syndrome, Peutz-Jeghers syndrome, neurofibromatosis, cleidocranial dysplasia and ectodermal dysplasias were also frequently mentioned. This suggests significant knowledge of these diseases among OM/OP specialists. The association between the presence of oral alterations and the mentioned pathologies aligns with previous case reports and observational studies (5, 9, 20, 31, 46–48). It is necessary for OM/OP specialists to have knowledge about the phenotypic characteristics of rare genetic diseases. As early diagnosis will ensure appropriate and timely multidisciplinary referrals (49). Furthermore, early identification of syndromes such as Gorlin-Goltz (nevoid basal cell carcinoma syndrome), Gardner, Peutz-Jeghers, and Cowden is crucial for guiding early diagnosis and treatment planning, especially for neoplasms associated with these conditions (50–52).

This study provides important evidence about the knowledge and practices of OM/OP specialists concerning medical genetics, revealing a growing awareness of the importance of genetic diseases in dental health. However, some limitations should be considered. First, the sample size, though representative of Brazilian regions, may limit the generalizability of findings to other contexts. Second, the inclusion criteria were based on membership in the SOBEP, which aggregates specialists in both OM and OP. Importantly, we did not stratify participants by subspecialty, which could influence responses. This limitation precluded stratified analyses and highlights the need for future studies to differentiate these groups. The association of time since PhD suggests that advanced training may increase diagnostic suspicion, but the overall lack of association highlights potential gaps in standardized genetics education in OM/OP training programs, rather than individual experience alone.

Finally, regional disparities in Brazil's healthcare infrastructure, such as uneven access to genetic testing and centralized specialist services, may have influenced participants’ practical engagement with genetic diagnostics, a factor not fully captured in this study. Despite these limitations, the findings provide critical insight into the gap between theoretical and practical knowledge in medical genetics among OM/OP specialists. They underscore the urgency of educational interventions that integrate clinical genetics into dental curricula and foster interdisciplinary collaboration. Future research should incorporate subspecialty-specific analyses (Oral Medicine vs. Oral Pathology) and explore systemic barriers, such as uneven resource distribution, to optimize care for patients with rare genetic diseases.

There is inadequate integration between Medical Genetics and OM/OP, with a lack of professionals and multidisciplinary teams being cited by practitioners as limiting factors. However, there is a widespread recognition among professionals of the presence of oral alterations as indicators of genetic diseases. The creation of policies that support the formation of multidisciplinary teams, the development of specialized genetic clinical services, and interprofessional training in the field could facilitate the adoption of a comprehensive and integrated approach to care.

## Data Availability

The raw data supporting the conclusions of this article will be made available by the authors, without undue reservation.
